# Phototoxicity of brightfield live-cell imaging on murine ovarian follicles

**DOI:** 10.1007/s00404-026-08515-y

**Published:** 2026-07-03

**Authors:** Marie Stadter, Ralf Dittrich, Lothar Häberle, Laura Lotz, Stefanie Brey, Nathalie Bleisinger, Benjamin Schmid, Matthias W. Beckmann, Anna K. Dietl

**Affiliations:** 1 Department of Obstetrics and Gynecology, Universitätsklinikum Erlangen, Friedrich-Alexander-Universität Erlangen-Nürnberg, Universitätsstraße 21–23, 91054 Erlangen, Germany; 2 Optical Imaging Centre Erlangen (OICE), Friedrich-Alexander-Universität Erlangen-Nürnberg, Cauerstraße 3, 91058 Erlangen, Germany; 3 Department of Biology, Division of Genetics, Nikolaus-Fiebiger-Center for Molecular Medicine, Friedrich-Alexander-Universität Erlangen-Nürnberg (FAU), Glückstraße 6, 91054 Erlangen, Germany; 4 MVZ Kinderwunschzentrum, Emailfabrikstraße 15, 92224 Amberg, Germany

**Keywords:** Time-lapse, Artificial ovary, Fertility preservation, Light exposure

## Abstract

**Purpose:**

Within the female reproductive tract, fertilization and embryo development occur in the complete absence of light. Light exposure during the in vitro manipulation of ovarian follicles negatively affects follicle viability. Laboratory conditions involve exposure to varying wavelengths, intensities, and light sources.

**Methods:**

In our study, we observed the effect of recurring light exposure on murine ovarian follicle viability subsequently assessed by evaluating increase in diameter, changes in morphology, and results from the LIVE/DEAD assay. A time-lapse approach allowed for effective observation of follicle interactions and viability, with no relevant movement or loss of focus throughout the imaging period.

**Results:**

However, follicles exposed to light showed a significant increase in granulosa cell death, as indicated by the LIVE/DEAD assay. No significant difference was observed in regard to morphology.

**Conclusion:**

Brightfield time-lapse provides a valuable tool for identifying early predictive signs and key milestones in follicle development, which may enhance in vitro follicle culture techniques and improve patient treatment outcomes, but simultaneously carries a risk of phototoxicity that must be considered when applying this method.

**Supplementary Information:**

The online version contains supplementary material available at 10.1007/s00404-026-08515-y.

## What does this study add to the clinical work?

**Table:** 

This study highlights the importance of minimizing light exposure during in vitro follicle culture to preserve follicle viability, thereby contributing to the optimization of artificial ovary technologies. These findings support the development of tumor cell-free scaffolds containing isolated ovarian follicles as a fertility preservation strategy for patients in whom ovarian tissue transplantation is contraindicated because of the risk of malignant cell contamination.	

## Introduction

A major cause of infertility in girls and young women is an oncological treatment during early adolescence. With rising survival rates, attention is increasingly focused on life after cancer, particularly the preservation of the patients’ fertility prior to gonadotoxic therapies. The two main options for fertility preservation before cytotoxic treatment—the cryopreservation of oocytes or, alternatively, the cryoconservation of ovarian tissue—do not prove feasible for every patient (e.g., because of the risk of transferring malignant cells together with the ovarian tissue) [1].

To address this problem, ovarian follicles are subject of ongoing experimental research. The goal is to develop an artificial ovary in which follicles can be cultured in vitro (or even in vivo in the patient herself) until the egg cells are mature and fertile. Recent studies have demonstrated promising results with the use of electrospun synthetic scaffolds from polycaprolactone (PCL)/gelatin polymers, hydrogels from natural polymers like fibrin and alginate, or decellularized extracellular matrix [2, 3]. Notably, in mice, a 3D-printed gelatin scaffold seeded with murine secondary follicles and retransplanted into an ovariectomized mouse, successfully supported folliculogenesis, leading to the birth of living offspring [4].

For optimizing the in vitro follicle culture and assessing the effects of different substances and/or conditions on follicular survival, live-cell imaging is essential. Using a light microscope suggests itself as the obvious approach for checking the follicle morphology. But especially while monitoring the follicular development over a longer time, frequent removal of the culture dishes from the incubator causes various, undesirable changes to the environment. These issues can be mitigated by using time-lapse microscopy within an enclosed chamber that maintains stable CO_2_ saturation, temperature, and humidity.

Current research utilizing confocal microscopy, with a high photon dose, to study follicular dynamics is limited to ovarian tissue [5–7]. Clinical experiences also exist for using confocal time-lapse imaging as part of assisted reproductive technology (ART) to check the quality of oocytes and embryos before transfer [8]. However, to date, no studies have employed time-lapse imaging to investigate follicular dynamics within artificial ovaries. In addition, the existing imaging studies ignore the effects on cell viability. As Cole [9] aptly put it: “While live-cell imaging is well appreciated, the need to ensure cellular health is not.”

Other research on the effects of light, particularly of short wavelengths, suggests that it causes DNA changes and cell damage in oocytes and embryos. However, the overall results are heterogeneous [10].

Since follicles are not exposed to light in the female body either, they may not have sufficient ability to protect themselves against it as well. To the best of our knowledge, these effects of in vitro light exposure on ovarian follicles have not been subject to research thus far. They are exposed to various types of light in laboratory work: natural light through the windows, lamp light of the laminar flow hood for seeding and medium change, and microscope light while monitoring development in the scaffold [11]. While the ultimate clinical application of in vitro follicle culture for fertility preservation remains experimental and not yet feasible, studying the effects of environmental factors such as light exposure provides essential knowledge for optimizing culture conditions and ensuring cellular health in future translational approaches.

This study evaluated the feasibility of using brightfield time-lapse microscopy as a method to investigate the development, interaction, and migration behavior of living follicles cultured in scaffolds in vitro. Additionally, the study examined the effects of repeated light exposure from microscopy on follicular growth, morphology, and viability. Although brightfield microscopy is widely used, as it offers a lower photon dose compared to confocal imaging, its application in long-term time-lapse imaging of murine ovarian follicles within culture scaffolds has not been systematically assessed, and the potential phototoxic effects on follicle viability remain largely unexplored.

## Materials and methods

### Animals

Ten 4-week-old female mice (B6/N) were acquired from Janvier Labs (Le Genest Saint Isle, France) and housed under controlled environmental conditions with free access to water and food. Illumination was provided from 06:00 to 18:00 h. Mice were accommodated at a stocking density of 2–4 animals per cage (floor area 530 cm^2^) in a 21 °C, humidity-controlled care facility at University of Erlangen-Nuremberg, accredited by the Association for Assessment and Accreditation of Laboratory Animal Care. The animals were also used for breeding colony maintenance and had undergone superovulation using pregnant mare serum gonadotropin (PMSG) followed by human chorionic gonadotropin (hCG) according to institutional protocols. The mice were sacrificed by controlled release of CO_2_ with every effort made to minimize suffering. For the experiments, 20 ovaries were used. After removal, ovaries were transported in Dulbeccos Phosphate Buffered Saline (DPBS, anprotec, Bruckberg, Germany) and cooled at 4 °C as suggested by Raffel et al. [2].

### Enzymatic digestion of ovarian tissue

To release the follicles from the surrounding stroma, the ovaries were digested by collagenase (type IV; Sigma-Aldrich, Saint Louis, Missouri, USA). Therefore, 1 ml of a freshly prepared collagenase/DPBS-solution (DPBS with calcium; Sigma-Aldrich, Saint Louis, Missouri, USA) with a concentration of 1.5 mg/ml was added to ten ovaries, respectively. The ovaries were incubated for 25 min at 37 °C in a water bath. For a more efficient digestion, the ovaries were pipetted up and down 10 times after 5, 10, and 20 min using a battery pipetting device (PIPETGIRL; Integra Biosciences, Zizers, Switzerland) and a 2 ml serological pipette (Costar Stripette; Corning, NY, USA). The collagenase reaction was stopped by adding cold DPBS (without calcium; Sigma-Aldrich, Saint Louis, Missouri, USA). The dissociated ovaries were put into a 90 mm petri dish (NUNC IVF DISH for in vitro fertilization; Thermo Fisher Scientific Inc., Waltham, Massachusetts, USA).

### Follicle isolation, selecting suitable follicles for culture, and seeding into the cell culture insert

Intact primary and secondary follicles were collected with an upright transmitted light stereomicroscope (Wild M8; Wild Heerbrugg, Switzerland) and picked up using a 3 µl precision micropipette with a 275 µm tip (The STRIPPER, STRIPPER Tips, 275 µm; Origio, CooperSurgical, Inc., Trumbull, Connecticut, USA). Although hormonal stimulation increases the proportion of growing follicles, antral follicles were excluded during manual isolation to obtain a more homogeneous population of primary and secondary follicles for culture and analysis. For identification of high-quality follicles, the following aspects, similar to those employed by Shikanov et al.[12] and Converse et al. [13], were taken into account: (1) presence of a non-ruptured granulosa cell layer with an intact basal membrane, (2) a clear, round oocyte, and (3) no separation between granulosa cells and oocyte. The follicles were gently washed and then 30 follicles were placed in each insert, as culture of multiple follicles is known to improve the survival of late primary and early secondary follicles [14]. All follicles in this study were manually allocated in groups of 10 follicles to the experimental groups with the aim to build two comparable groups regarding follicle size. To check this, the follicles were subsequently measured after seeding into the scaffold.

Each culture insert contained 30 follicles. In total, 12 cultures were established. The experiment was performed in three experimental runs (E1-E3). Each experimental run comprised four cultures and was subdivided into two subgroups (Sg1 and Sg2). Within each subgroup, one culture was assigned to the light-exposed group and one culture to the control group. Consequently, each experimental run included two light-exposed cultures and two control cultures. Across all experiments, six cultures (180 follicles) were analyzed in the light-exposed group and six cultures (180 follicles) in the control group.

**Fig. 1:**
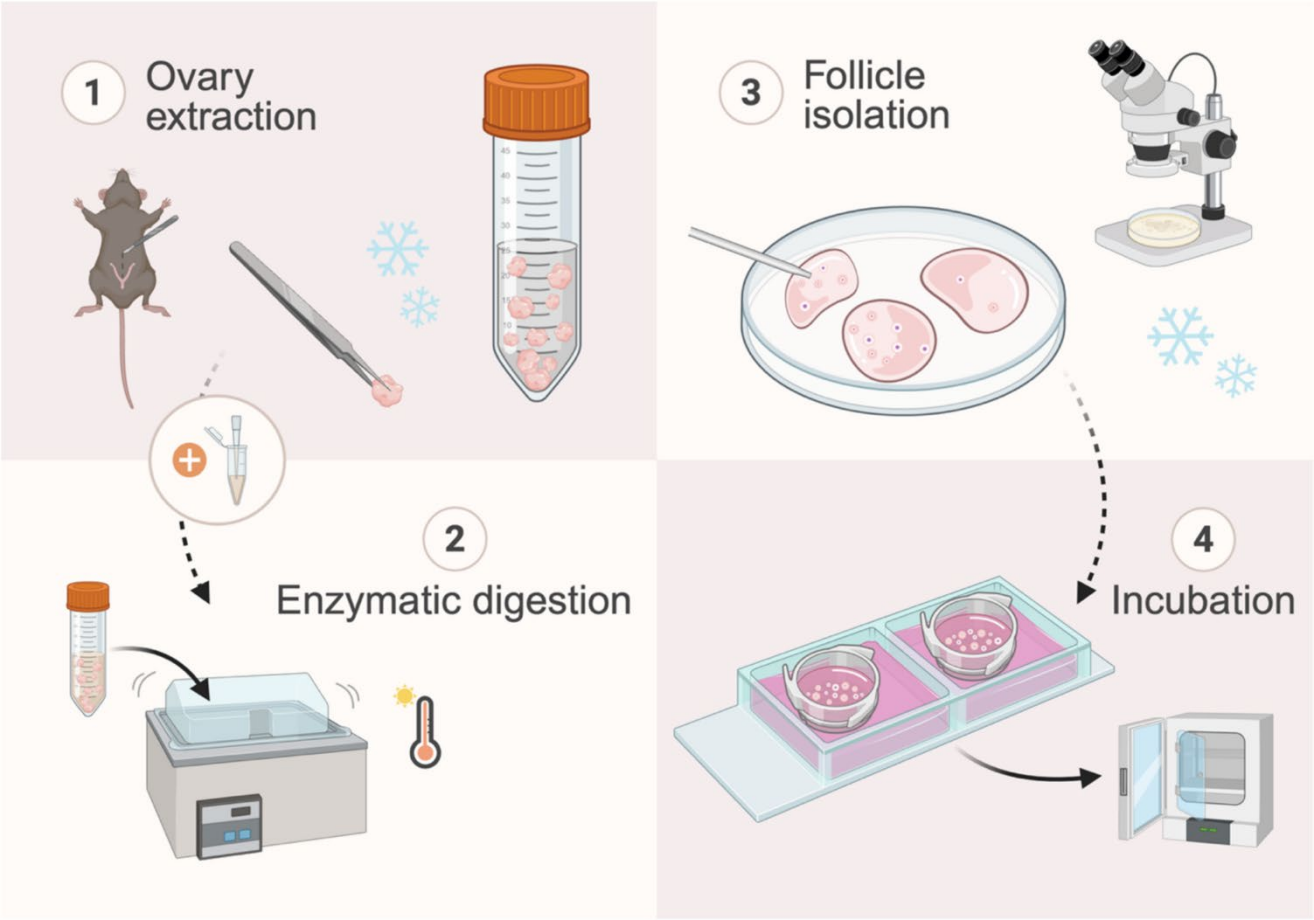
Follicle collection workflow. (1) Extraction of the ovaries and transportation at 4 °C. (2) Enzymatic tissue digestion with collagenase at 37 °C in a water bath. (3) Microscopic isolation of the follicles using a precision micropipette. (4) Transfer of the follicles into a cell culture insert and incubation under standard culture conditions. Created in BioRender. Stadter, M. (2025) https://BioRender.com/h1v1vsn

A sufficient amount of medium, a modified version of the serum-free medium of Thomas et al. [15], was freshly prepared for each cultivation. It was consisting of McCoy’s 5a medium (Gibco, Thermo Fisher Scientific Inc., Waltham, Massachusetts, USA) with 20 mM HEPES buffer solution (Gibco, Thermo Fisher Scientific Inc., Waltham, Massachusetts, USA), 3 mM L-glutamine (Gibco, Thermo Fisher Scientific Inc., Waltham, Massachusetts, USA), 0.1% human serum albumin (SAGE In Vitro Fertilization, CooperSurgical, Inc., Trumbull, Connecticut, USA), 0.1 mg/ml each penicillin and streptomycin (Sigma-Aldrich, Saint Louis, Missouri, USA), 2.5 µg/ml amphothericin B (Gibco, Thermo Fisher Scientific Inc., Waltham, Massachusetts, USA), 8 µg/ml insulin, 4.4 µg/ml transferrin, 4 ng/ml selenium (ITS solution; Sigma-Aldrich, Saint Louis, Missouri, USA), 50 µg/ml L-ascorbic acid (Sigma-Aldrich, Saint Louis, Missouri, USA), and 0.272 IU r-FSH (Gonal-f; Merck, Darmstadt, Germany), as recommended by Converse et al.[13]. L-ascorbic acid was added directly before use of the medium in order to prevent a premature oxidation to dehydroascorbic acid [16]. 1.8 ml of the culture medium were put in each well and 0.6 ml were added per insert. After 48 h, half of the medium was replaced with new medium that had been incubated overnight. To minimize further fluctuations in pH and temperature as well as light exposure, the time outside the incubator was limited to less than 5 min for each step (Fig. 1).

### Experimental design phototoxicity: the impact of light on follicles

To investigate the effects of repeated light exposure on follicles, the control group and the exposure group were each incubated for 48 h in a two-well coverslide with glass bottom (Ibidi, Gräfelfing, Germany), in two polytetrafluoroethylene (PTFE) inserts (PICM01250; Millipore, Merck, Darmstadt, Germany). The scaffolds with a biopore membrane out of hydrophilic PTFE are common commercial cell culture inserts for follicle and ovary culture. They are compatible with fluorescent staining and made for live-cell viewing.

After changing the medium, both groups were transferred to the chamber of the microscope (Incubator i8 with CO_2_ lid KM; PeCon, Erbach, Germany) to rule out potential differences due to incubation. Only the coverslide of the exposure group was inserted in the slide holder, the “dark” control group was permanently out of the light beam. The follicles were incubated for 24 h in the microscope chamber at 37 °C and 5% CO_2_. To prevent excessive evaporation of the medium, the lid of the coverslip was closed, and falcon lids filled with sterile water were set up for high humidity. All components of the microscope and the specimens were preheated sufficiently to prevent a focus shift.

In the study group, an image of every follicle was taken every 15 min with a THUNDER Imager 3D Live Cell (Leica Microsystems GmbH, Wetzlar, Germany), which is especially developed for long-term low-damage live-cell imaging. This high-frequency interval was chosen as a “worst-case-scenario”, to maximize potential light exposure and induce measurable phototoxic effects, but is also in line with previously published time-lapse protocols [17]. For this, a monochromatic Leica DFC9000 sCMOS Camera and a 10 × objective with a numeric aperture of 0.32 was used (HC PL FLUOTAR 10x/0.32 PH1). The exposure time was 20 ms at an intensity of 130, aperture of 1, and TL-Fld. of 25. Over 24 h, the follicles were exposed to light for a total of 19.4 s (97 timepoints × 0.02 s × 10 positions).

Morphology of each follicle was investigated afterward, and to compare the viability of the follicles, a LIVE/DEAD assay of each follicle was performed. Furthermore, follicle diameter was measured before and after the incubation/imaging period.

The experiments were performed in three independent runs using follicles isolated from different sets of ovaries. Despite standardized isolation and culture procedures, biological variability between donor animals and ovaries, including differences in follicle size distribution, developmental stage, and follicular microenvironment, as well as minor technical variability during isolation and culture, were expected to contribute to inter-experimental differences. Therefore, each experimental run was considered separately during statistical analysis.

### Assessments of follicle survival and growth

**Fig. 2:**
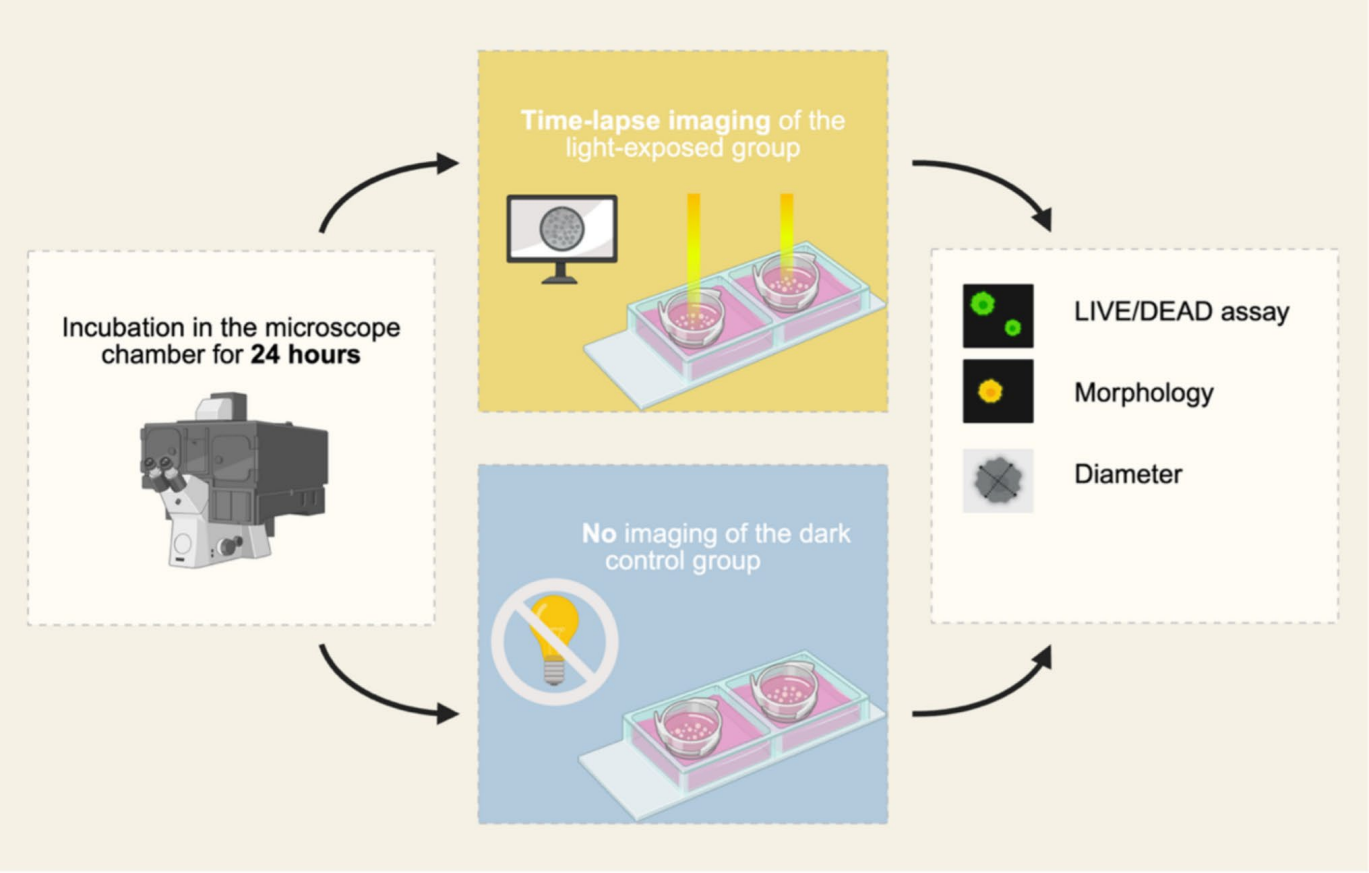
Experimental design phototoxicity: Both groups were incubated in the microscope chamber for 24 h. Time-lapse imaging was only performed on the light-exposed group. Afterward, the follicles were assessed regarding viability, morphology, and diameter. Created in BioRender, adapted from Stadter, M. (2025) https://BioRender.com/enpm33o

The LIVE/DEAD Viability/Cytotoxicity Kit (Invitrogen, Thermo Fisher Scientific Inc., Waltham, Massachusetts, USA) was used for determining the follicle viability as established by Maltaris et al. [18]. Part of the assay was calcein-AM, a non-fluorescent molecule taken up only by living cells. The green fluorescent calcein, with an emission of ~ 517 nm, is produced by intracellular esterases that cleave off the acetoxymethyl group. The red ethidium homodimer-1 (Ethd-1) was used as a marker for dying cells. Ethd-1 enters the interior of a defective cell due to the increasing permeability of a fragile cell wall, binds to nucleic acids, and subsequently emits ~ 617 nm. The assay was performed as follows: Per milliliter of DPBS (with Ca and Mg; Sigma-Aldrich, Saint Louis, Missouri, USA), 0.25 µl of 4 mM calcein and 0.5 µl of 2 mM Ethd-1 were added. For staining, the medium was removed from the cell culture inserts to prevent fluorescence of the environment. Next, the staining solution was gently run over the edge of the well and insert until the 30 follicles were sufficiently covered. The follicles were incubated for 30 min at 37 °C in the dark, and then, images were taken (Fig. 2). Three follicles in each group got lost to an end of 177 manually examined follicles.

**Fig. 3:**
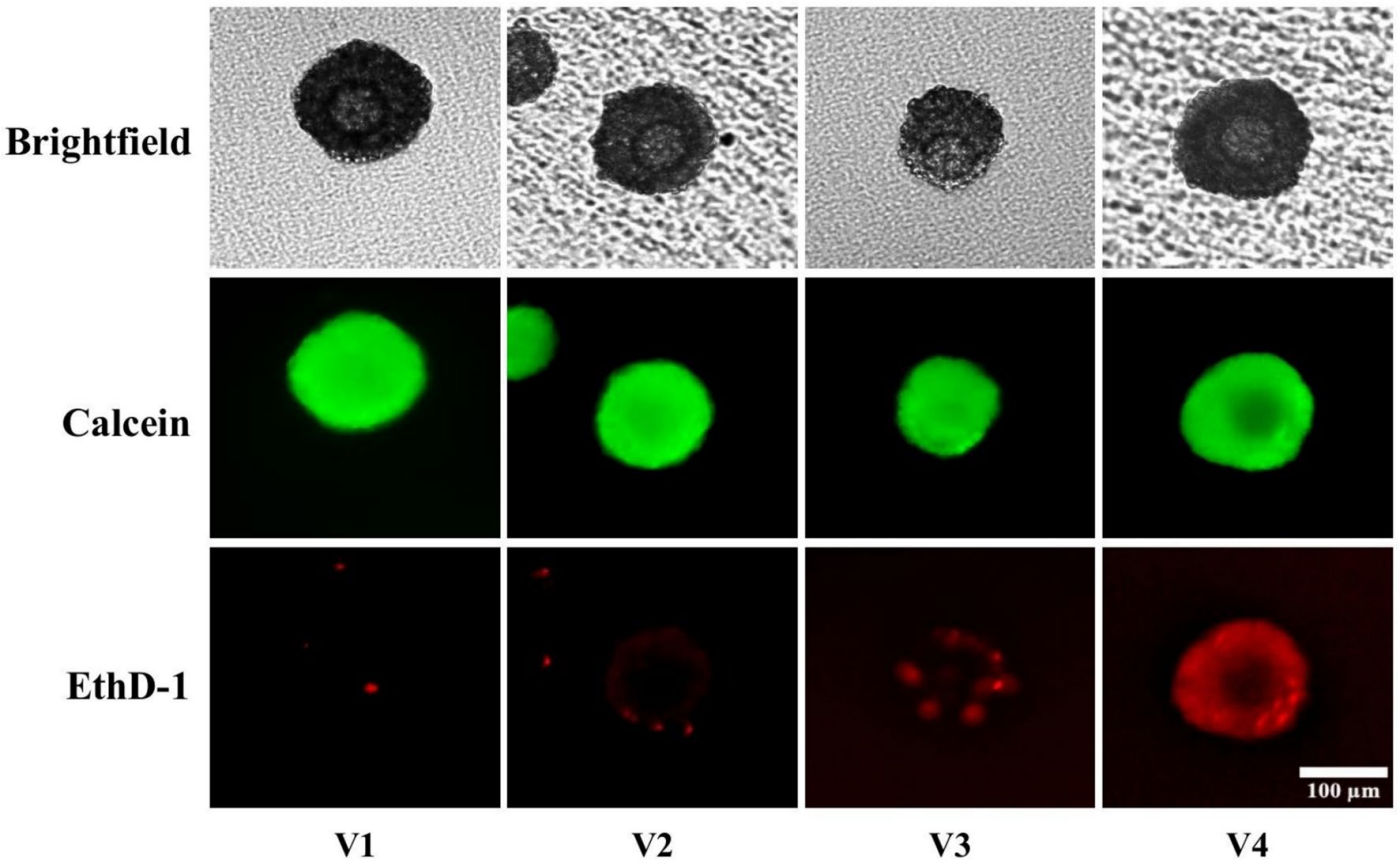
Classification of follicles into four groups based on the number of damaged granulosa cells in the LIVE/DEAD assay: V1 (living follicles with living oocyte and < 1% damaged GC), V2 (1–10% damaged GC), V3 (10–50% dead GC), and V4 (> 50% GC or dead oocyte). The same follicle is shown in brightfield, stained with calcein (indicator of living cells) and EthD-1 (dead cells)

Based on its viability, each follicle was visually assigned to one of the following four groups, similar to those specified by Dolmans et al. [19] and Fattahi et al. [20]: V1 for living follicles with a viable oocyte and < 1% damaged granulosa cells (GC); V2 for minimally damaged follicles with 1–10% dead GC; V3 for moderately damaged follicles with 10–50% dead GC; and V4 for dead follicles with a dead oocyte or > 50% dead GC (Fig. 3).

**Fig. 4:**
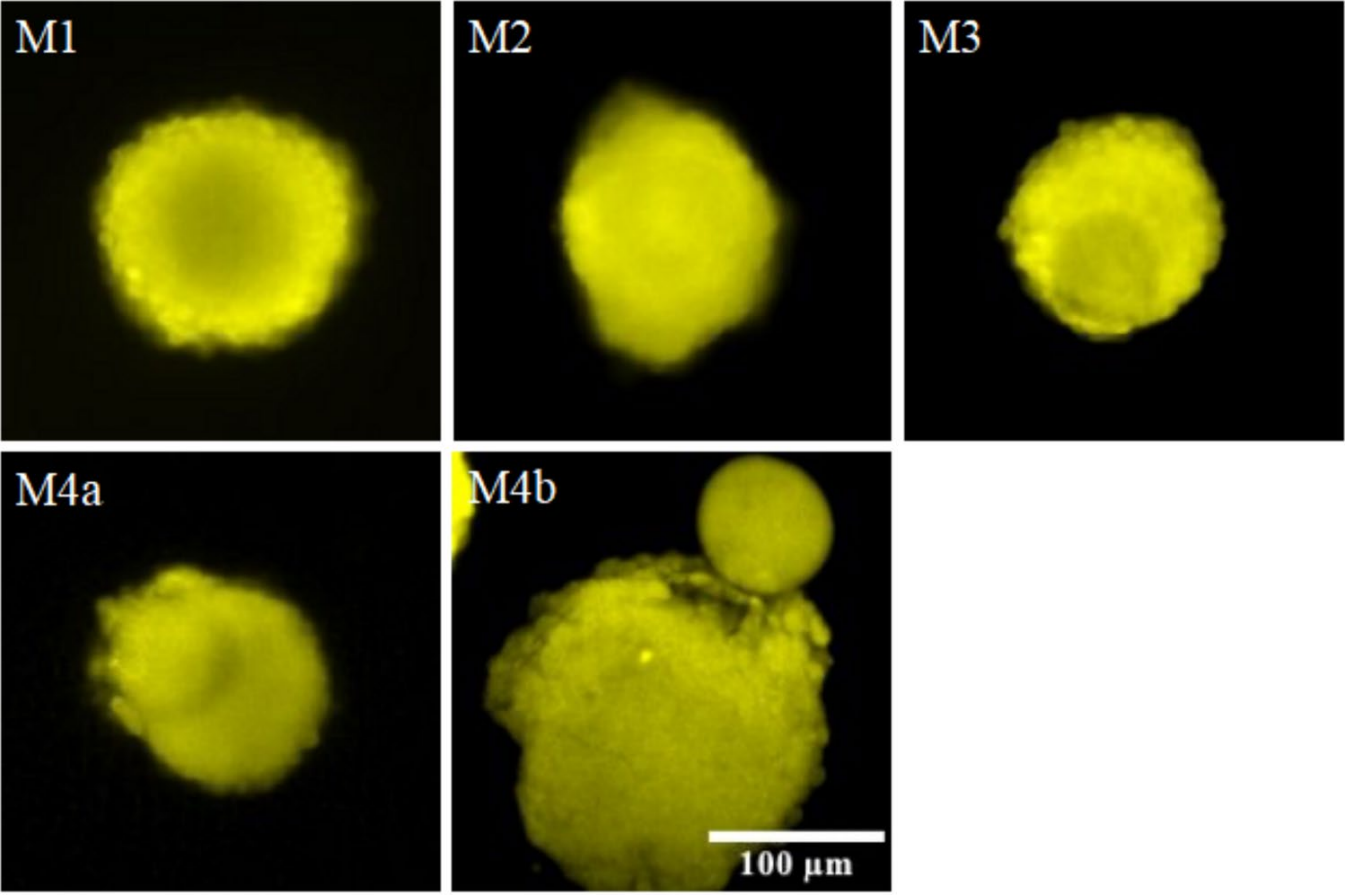
The follicles were assessed regarding their morphology and classified into five groups: M1 (spherical shape with intact granulosa cell layer), M2 (irregular shape with intact GC layer), M3 (irregular shape with < 10% loss of GC), M4a (totally atypical shape with 10–50% loss of GC), and M4b (extruded oocyte)

For evaluation of morphology, follicle shape as well as intactness of the granulosa cell layer were examined. Each of the 30 follicles per well was visually classified according to the following four categories of Dolmans et al. [19]: M1 spherical shape with intact granulosa cell layer; M2 irregular shape with intact GC layer; M3 irregular shape with < 10% loss of GC; M4a totally atypical shape with 10–50% loss of GC or M4b an extruded oocyte (Fig. 4).

To avoid double counting, a spiral image of the entire insert was taken before every step. The interactive overview allowed locating the follicles in the scaffold and imaging each follicle only once. To determine the follicle diameter, images of all follicles were taken at the area of contact with the scaffold. Measurement of follicle diameter was carried out in Fiji by taking two perpendicular measurements through each follicle from basal membrane to basal membrane and calculating the average.

### Statistical methods

Follicle diameters are summarized by the mean and standard deviation (SD), median and interquartile range (1st and 3rd quartile), and minimum and maximum. Summary statistics are presented separately for each group (light-exposed group, control group) and each experiment (E1 Sg1, E1 Sg2, E2 Sg1, E2 Sg2, E3 Sg2).

The growth of the follicles was analyzed only descriptively, because the repeated measurements of a follicle before and after light exposure could not be uniquely assigned to the respective follicle. In the control group—due to lack of continuous observation and chances in morphology and position of the scaffold—it was not reliably possible to find the same follicle again in the scaffold.

Both groups were compared with regard to morphology (ordinal categories M1–M4) using a multivariable proportional odds logistic regression analysis with morphology as the outcome, group as the predictor variable of interest and experiment as the covariate. The null hypothesis that both groups are equal with regard to morphology was tested using a likelihood ratio test. The adjusted proportional odds ratio (OR) with 95% confidence interval (CI) for light-exposed group versus control group is shown. OR < 1 means that the shapes of the follicles in the light-exposed group were more regular than those in the control group, whereas OR > 1 means that the shapes of the follicles in the light group were more atypical than those in the control group.

A similar analysis was carried out for viability (ordinal categories V1–V4).

Experiment was included as covariate in the analyses to address the fact that results differ across experiments (for details, see Sect. "Experimental design phototoxicity: the impact of light on follicles"). A simple proportional odds logistic regression analysis without any covariates corresponds to a Wilcoxon rank-sum test for ordinal categories.

All of the statistical tests were two-sided, and a *P* value < 0.05 was regarded as statistically significant. To address the problem of multiple testing, the *P* values of the statistical tests described above (two tests) were corrected using the Bonferroni method. Both raw and Bonferroni corrected *P* values were reported.

## Results

### Feasibility of a time-lapse

**Fig. 5:**
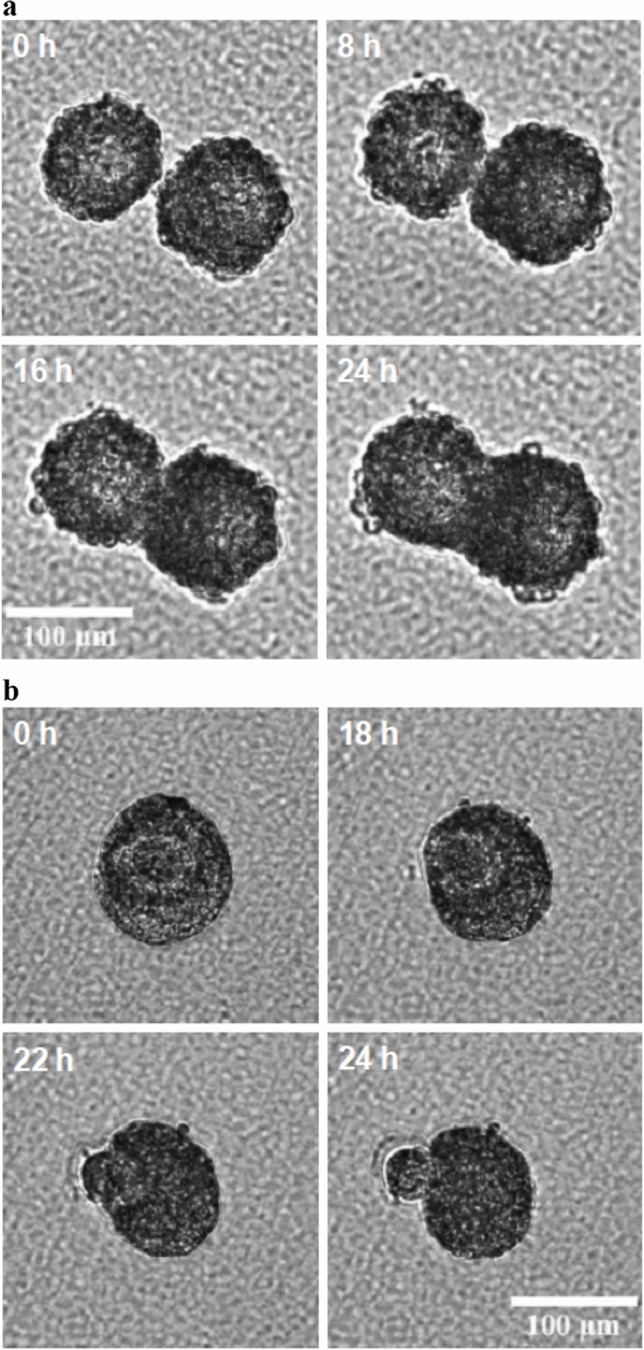
**a** Four images of a 24-h time-lapse showing two follicles finding each other and growing together. A full video is part of the supplementary materials. **b** The same follicle shown on four selected time points of a 24-h time-lapse. The oocyte with a dark cytoplasm already stands out at 0 h. At 18 h, the follicle starts visibly shrinking, and after 24 h, the oocyte gets extruded. A full video is part of the supplementary materials

The time-lapse approach allowed for effective observation of follicle interactions and viability, with no significant movement or loss of focus throughout the imaging period. In particular, the following behaviors were observed: The follicles did not migrate, nor did they frequently interact with the PTFE membrane. When two follicles were close to each other, they grew together [21] (Fig. 5a).

The supplementary video (video 1) demonstrates that follicles positioned in proximity grow together over time, providing a clear visualization of this interaction.

Additionally, dying of follicles was characterized by growth arrest or losing connection with the oocyte Fig. 5b.

A full video is provided as part of the supplementary materials, demonstrating the key stages of the process outlined below: The oocyte with a dark cytoplasm already stands out at 0 h. At 18 h, the follicle starts visibly shrinking, and after 24 h, the oocyte gets extruded (video 2). The various ways of dying (losing oocyte connection, shrinking of the follicle, falling apart, and optical features of apoptosis [22]) can be effectively studied with this method. Moreover, the time-lapse approach could prove feasible in further research on the oocyte rescue as described by Hornick et al. [14]. Finding predictive warning signs like a dark oocyte is possible as well.

### Follicle growth

**Fig. 6:**
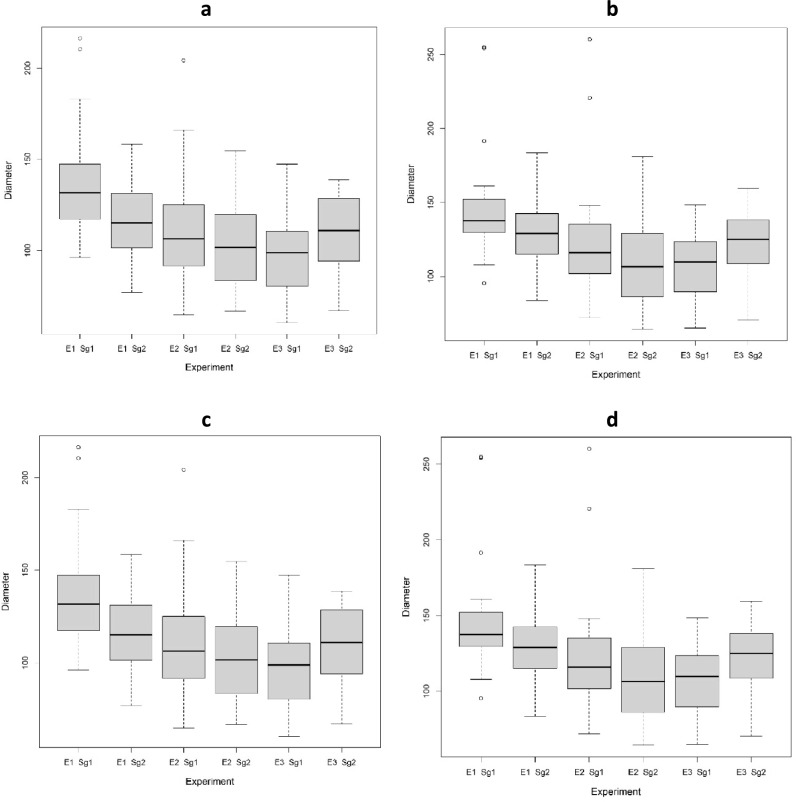
Follicle diameter (in μm) of the light-exposed group before (**6a**) and after light exposition (**6b**), and follicle diameter (in μm) of the control group before (**6c**) and after incubation in the microscope chamber (**6d**), showing boxplots for each experiment

The average follicle diameter differs between experiments (Table 1, Fig. 6). The initial average diameter in the light-exposed group ranged from 98.7 (SD 21.6 µm) to 134.5 µm (SD 30.3 µm), and it ranged from 100.2 (SD 19.5 µm) to 120.9 µm (SD 40.0 µm) in the control group (Table 1). After light exposure, the average diameter ranged from 108.5 (SD 22.3) to 144.8 µm (SD 35.1) in the light-exposed group and from 109.2 (SD 26.8 µm) to 141.8 µm (SD 54.4 µm) in the control group (Table 1).

**Table 1:** Follicle diameter (in μm) of the light-exposed group and the control group before and after light exposition and incubation in the microscope chamber, respectively, showing summary statistics for each experiment

Experiment	Statistic	Light-exposed group		Control group	
		Before	After	Before	After
E1 Sg1	mean (SD)	134.5 (30.3)	144.8 (35.1)	120.9 (40.0)	141.8 (54.4)
	median (IQR)	131.7 (117.6, 147)	137.5 (130, 151.1)	112.5 (98, 132.4)	128.9 (111.8, 140.3)
	(min, max)	(96.1, 216.3)	(95.2, 255.0)	(62.8, 272.1)	(72.6, 333.0)
E1 Sg2	mean (SD)	117.9 (21.0)	131.3 (23.5)	113.3 (18.5)	121.2 (28.9)
	median (IQR)	115.2 (101.6, 130.8)	129 (115.4, 142.4)	112.5 (100.1, 119.4)	115.4 (106.8, 127.5)
	(min, max)	(77.0, 158.4)	(83.8, 183.5)	(79.0, 159.4)	(75.7, 206.5)
E2 Sg1	mean (SD)	110.3 (29.8)	123.3 (38.2)	103.8 (24.0)	109.5 (28.5)
	median (IQR)	106.5 (92.5, 124.2)	116.0 (102.0, 135.1)	103.2 (83.1, 122.1)	112.0 (87.0, 128.8)
	(min, max)	(64.8, 204.3)	(72.1, 260.2)	(67.4, 159.7)	(73.5, 178.0)
E2 Sg2	mean (SD)	103.8 (23.3)	109.9 (26.8)	100.2 (19.5)	109.2 (26.8)
	median (IQR)	101.7 (84.1, 118.7)	106.5 (87.7, 128.7)	101.4 (85.5, 114.1)	106.8 (88.4, 125.3)
	(min, max)	(66.8, 154.7)	(64.4, 181.2)	(63.7, 134.9)	(71.4, 165.9)
E3 Sg1	mean (SD)	98.7 (21.6)	108.5 (22.3)	109.6 (17.0)	118.6 (15.4)
	median (IQR)	98.8 (81, 110.4)	109.8 (90.5, 122.7)	108.2 (95.1, 117.5)	119.5 (108.5, 126.5)
	(min, max)	(60.4, 147.5)	(65.1, 148.5)	(84.3, 154.3)	(83.3, 159.9)
E3 Sg2	mean (SD)	109.5 (19.1)	123.3 (20.1)	110.0 (19.3)	119.7 (20.0)
	median (IQR)	111 (94.6, 127.9)	125.0 (109.2, 137.2)	108.3 (99, 124.8)	123.5 (104.4, 135)
	(min, max)	(67.0, 138.8)	(70.5, 159.5)	(67.4, 142.1)	(78.4, 157.3)
*overall*	mean (SD)	112.4 (26.8)	123.5 (30.7)	109.6 (25.0)	119.9 (32.8)
	median (IQR)	108.7 (95.6, 128.8)	123.2 (107, 138.4)	108.2 (95.1, 121)	116.8 (103.5, 132.5)
	(min, max)	(60.4, 216.3)	(64.4, 260.2)	(62.8, 272.1)	(71.4, 333.0)

*E* experiment*, Sg* subgroup, *SD* standard deviation, *IQR* interquartile range

Overall, the initial average diameter in the light-exposed group was 112.4 µm (SD 26.8 µm) and slightly smaller in the dark control group with 109.6 µm (SD 25.0 µm). After light exposure, the light-exposed group grew to an average diameter of 123.5 µm (SD 30.7 µm). In the control group, the follicles grew to around 119.9 µm (SD 32.8) (Table 1). The increase in diameters was similar between light-exposed and control group (Table 1).

### Morphology

**Fig. 7:**
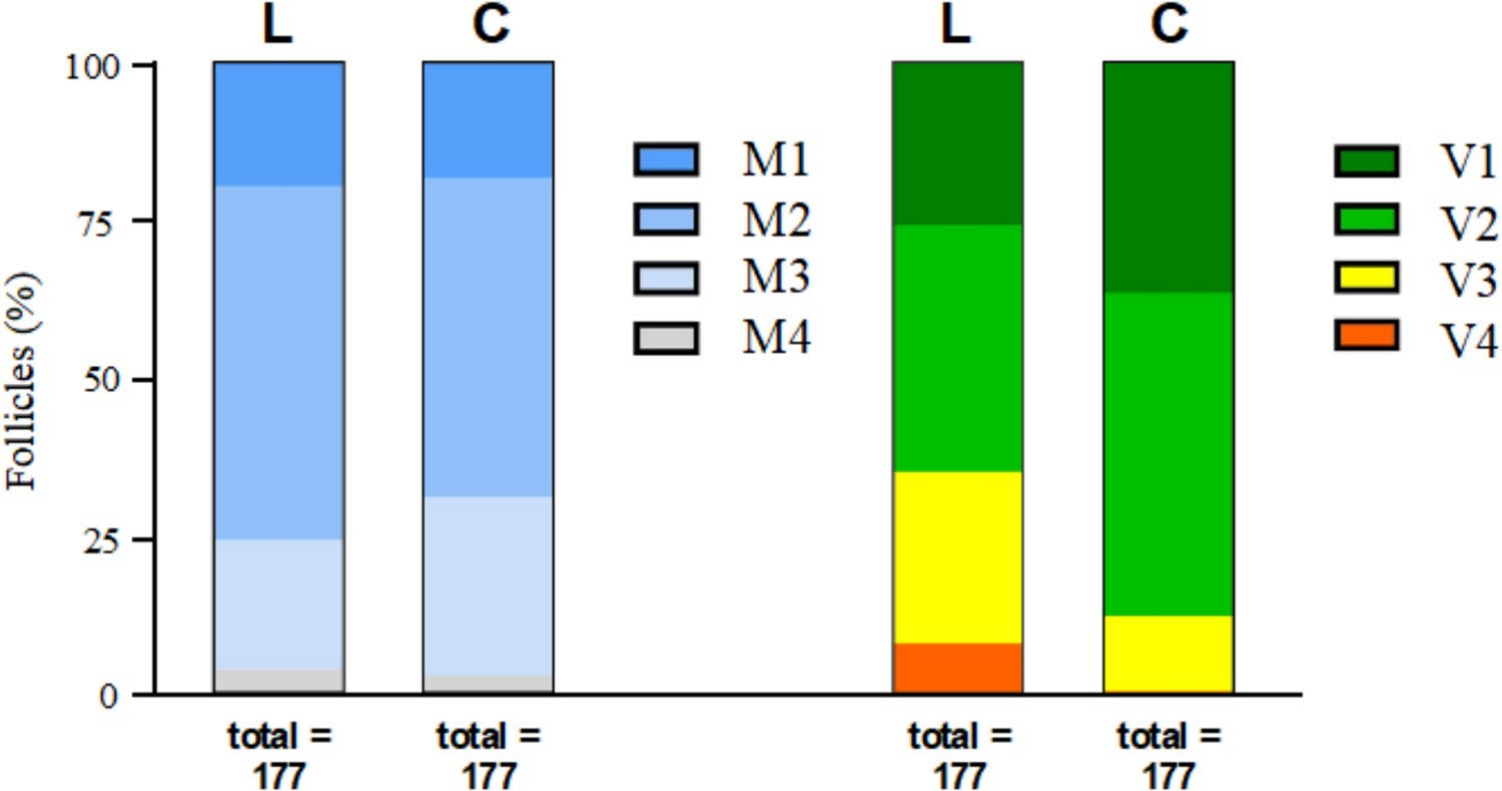
The follicles of the light-exposed group (L) and the control group (C) were individually classified regarding their morphology (on the left) and their viability (on the right). No significant difference was detected concerning the morphology. The differences regarding the viability were significant

In the light-exposed group, 19.77% of all of the follicles were classified as M1, 55.93% as M2, 20.34% as M3, and 4.00% as M4. In the control group, 18.64% were assigned to M1, 50.28% to M2, 28.25% to M3, and 2.82% to M4 (Table 2). No significant morphological differences were detected within the 24-h observation period (Fig. 7): The proportional OR for light versus control group was 0.81 (95% CI 0.55–1.21; raw *P* = 0.31, Bonferroni *P* = 0.61).

**Table 2:** Classification of each follicle into one of four groups regarding the morphology: M1–M4. The experiment was carried out 3 times with two subgroups (1 and 2) and 30 follicles per well

Morphology								
Experiment no	Light (*N* = 177)				Control (*N* = 177)			
	M1	M2	M3	M4	M1	M2	M3	M4
E1 Sg1	7	17	4	2	4	17	8	0
E1 Sg2	11	17	2	0	7	14	9	0
E2 Sg1	8	15	6	1	3	12	13	2
E2 Sg2	5	12	9	2	8	14	6	2
E3 Sg1	0	22	6	1	7	19	2	0
E3 Sg2	4	16	9	1	4	13	12	1
Total no. of follicles	35	99	36	7	33	89	50	5
%	19.77	55.93	20.34	4.00	18.64	50.28	28.25	2.82

### Viability

Regarding the viability score, that was assessed by classifying each follicle individually with the LIVE/DEAD assay, significantly more dead granulosa cells were found in light-exposed group. The follicles in the light-exposed group presented as V1 in 25.99% of cases, and as V2 in 38.89% compared to 36.72% as V1 and 50.85% as V2 in the control group. The follicles in moderate or bad condition made up 27.12% V3 and 7.91% V4 in the light-exposed group and 11.86% V3 and 0.56% V4 in the control group (Table 3). Viability was significantly poorer in the light-exposed group than in the control group: The proportional OR for light group versus control group was 2.44 (95% CI, 1.63–3.64; both raw and Bonferroni *P* < 0.0001).

**Table 3:** Classification of each follicle into one of four groups regarding the LIVE/DEAD assay: V1–V4. The experiment was carried out 3 times with two subgroups (1 and 2) and 30 follicles per well

Viability								
Experiment no	Light (*N* = 177)				Control (*N* = 177)			
	V1	V2	V3	V4	V1	V2	V3	V4
E1 Sg1	16	7	7	0	11	13	5	0
E1 Sg2	2	10	12	6	12	16	2	0
E2 Sg1	4	14	8	4	7	20	2	1
E2 Sg2	8	14	4	2	9	18	3	0
E3 Sg1	11	8	9	1	12	13	3	0
E3 Sg2	5	16	8	1	14	10	6	0
Total no. of follicles	46	69	48	14	65	90	21	1
%	25.99	38.98	27.12	7.91	36.72	50.85	11.86	0.56

## Discussion

In the present study, we investigated the effect of light on follicle growth, morphology, and viability. In the LIVE/DEAD assay, there have been significantly more dead granulosa cells in the light-exposed group compared to the control group. Due to the incubation in the same chamber and under the same conditions, other external factors which could potentially have led to cell damage (e.g., oxygen concentration, pH, temperature, and culture media [23]) are unlikely. Within the limitations of the experimental setup, the findings therefore suggest that light exposure during time-lapse imaging may contribute to increased granulosa cell injury.

In general, there are different ways light could have caused the cell damage, which can be divided into indirect and direct phototoxic effects. The main mechanism for indirect light-mediated damage in cell culture is by photooxidation of medium components [24, 25]. This can be prevented by carefully choosing and properly adapting the medium. For example, HEPES in the medium could have led to production of cytotoxic hydrogen peroxide when exposed to light [26]. Adding antioxidants, such as the ascorbic acid used in our experimental setup, can prevent the development of external reactive oxygen species (ROS) in the culture medium though [27]. Moreover, FSH, which was added to our medium, is known as being antiapoptotic in granulosa cells [28].

In addition, light can induce cell damage in a direct way by inflicting DNA-damage, localized heating, or generating ROS in the cell itself. These processes can contribute to oxidative stress, defined as an imbalance between pro- and antioxidative cellular systems. While oxidative stress has been associated with granulosa cell apoptosis in the previous studies, these mechanisms were not directly assessed in the present work and are therefore discussed as potential explanations [29]. Oxidative stress is already known to lead to stress-induced DNA-damage and apoptosis in granulosa cells and zygotes [30, 31]. Oxidative stress has been associated in the previous studies with granulosa cell apoptosis and impaired oocyte function; however, these mechanisms were not directly assessed in the present study [32, 33]. H_2_O_2_-mediated oxidative stress led to porcine granulosa cell apoptosis, decreased cell viability, and reduced follicular development [34].

Not only the DNA, but also the components of the cell membrane, like phospholipids and cholesterol, can be damaged by visible light-induced photooxidative stress [35]. Following loss of membrane integrity is a common viability assessment for phototoxicity [36] frequently done with a cell-impermeable staining that binds intracellular DNA [37]. For this purpose, the use of EthD-1 is established as a method for cell death in ovarian follicles [18]. Based on the LIVE/DEAD assay results, the findings suggest that particularly the outer granulosa cell layers may be more susceptible to light-associated damage. Although viability assessment was performed according to established classification systems, quantitative image analysis using ROI-based fluorescence intensity measurements may further increase precision and reduce potential observer bias in future studies.

Oocyte viability was included in the V4 classification (dead oocyte or > 50% damaged granulosa cells). However, specific functional parameters of oocyte quality, such as meiotic spindle integrity, mitochondrial activity, or DNA fragmentation, were not assessed in this study. The presence of extruded oocytes in both groups indicates that intrinsic degeneration processes occur independently of light exposure Nevertheless, the higher proportion of moderately and severely damaged follicles in the light-exposed group suggests that repeated or cumulative illumination may increase granulosa cell injury, which could potentially affect oocyte support functions.

In this work, no significant changes in the morphology between the light and the dark group were detected. Morphology alone may not fully reflect follicular viability, as follicles without growth over several days are typically considered non-viable despite preserved structural appearance [13]. The average increase in diameter was similar in both groups. It should be noted that primary and early secondary follicles are not expected to undergo substantial structural changes within 24 h. Longer culture periods may be required to determine whether early granulosa cell damage translates into structural degeneration. Future studies with longer observation windows are also warranted to determine whether the early increase in granulosa cell death translates into permanently impaired development or reduced oocyte quality.

As it could diminish the likelihood of achieving a successful pregnancy or live birth, it is important to take all reasonable safety precautions [38]. Bodis et al. [39] showed that working with light protection could expand the fertilization rate in intracytoplasmic sperm injection (ICSI) cycles and increase the numbers of following pregnancies. Also, Oh et al. [40] conclude that removing light as a stress factor improves embryo development. However, there are also limitations, as working in the dark can lead to additional errors on the part of staff [11]. The precise threshold of light exposure that can be considered ‘safe’ for follicle culture remains to be determined. Future studies employing dose–response experiments with varying light intensities and imaging intervals will be required to establish exposure guidelines for follicle imaging.

## Conclusion

In experimental research, the 24-h time-lapse with a THUNDER widefield microscope is a feasible method for studying follicle behavior, migration, and survival within a compatible scaffold. The approach is particularly suitable for observing the effects of various substances on follicles. It facilitates the identification of early predictive signs and relevant milestones in follicular development, contributing to the optimization of in vitro follicle culture and improving patient treatment strategies.

The findings of this study, in conjunction with existing research on light-induced damage in oocytes and embryos, suggest that exposure to light during microscopy should be minimized. To that end, it is recommended to use low light intensity, limit position-selection time, illumination duration, and incorporate extended dark recovery periods between exposures to reduce relevant factors for phototoxicity in live-cell imaging.

Alternative imaging schedules (e.g., lower frequency) may reduce phototoxicity further, and future studies are needed to systematically determine safe thresholds of exposure for follicle culture.

## Supplementary Information

Below is the link to the electronic supplementary material.

Supplementary file1 (AVI 1526 KB)

Supplementary file2 (AVI 1347 KB)

## Data Availability

The data that supports the findings of this study is available from the corresponding author, Ralf Dittrich, upon reasonable request.
